# Timing of urate-lowering therapy and risk of kidney failure and mortality in CKD: an application of the parametric G-formula

**DOI:** 10.1093/ckj/sfag103

**Published:** 2026-03-23

**Authors:** Ara Ko, Hyunman Sim, Soie Kwon, Seung Hyun Han, Ali Abu-Alfa, Dong Ki Kim, Chun Soo Lim, Jung Pyo Lee, Woojoo Lee

**Affiliations:** Department of Internal Medicine, Seoul National University College of Medicine, Seoul, Republic of Korea; Department of Internal Medicine, Seoul National University Hospital, Seoul, Republic of Korea; Department of Internal Medicine, Seoul National University Boramae Medical Center, Seoul, Republic of Korea; Department of Statistics, Jeonbuk National University, Jeonju, Republic of Korea; Department of Public Health Sciences, Graduate School of Public Health, Seoul National University, Seoul, Republic of Korea; Department of Internal Medicine, Chung-Ang University Heukseok Hospital, Seoul, Republic of Korea; Department of Internal Medicine, Chung-Ang University, Seoul, Republic of Korea; Department of Internal Medicine, Seoul National University College of Medicine, Seoul, Republic of Korea; Department of Internal Medicine, Seoul National University Boramae Medical Center, Seoul, Republic of Korea; Division of Nephrology and Hypertension, American University of Beirut, Beirut, Lebanon; Department of Internal Medicine, Seoul National University College of Medicine, Seoul, Republic of Korea; Department of Internal Medicine, Seoul National University Hospital, Seoul, Republic of Korea; Department of Internal Medicine, Seoul National University College of Medicine, Seoul, Republic of Korea; Department of Internal Medicine, Seoul National University Boramae Medical Center, Seoul, Republic of Korea; Department of Internal Medicine, Seoul National University College of Medicine, Seoul, Republic of Korea; Department of Internal Medicine, Seoul National University Boramae Medical Center, Seoul, Republic of Korea; Department of Public Health Sciences, Graduate School of Public Health, Seoul National University, Seoul, Republic of Korea

**Keywords:** causal inference, chronic kidney disease, G-formula, hyperuricemia, urate-lowering therapy

## Abstract

**Background:**

The optimal timing for initiating urate-lowering therapy (ULT) in chronic kidney disease (CKD) remains uncertain. Although randomized trials have provided robust evidence, their neutral findings and limited follow-up leave questions regarding whether earlier intervention improves long-term outcomes.

**Methods:**

Using two nationwide CKD cohorts in Korea (*n* = 27 260 and 9727), we applied the parametric g-formula to estimate simulated risks for end-stage kidney disease (ESKD) and all-cause mortality under alternative ULT thresholds. Data were divided into 6-month intervals, and six strategies were simulated: observed practice (reference), initiation when serum urate (sUA) reached ≥7, 8, 9, or 10 mg/dl, and no treatment. Models accounted for time-varying confounders affected by prior therapy, and cumulative risks were estimated over 22 years.

**Results:**

Deferring ULT to higher sUA thresholds or not initiating therapy was associated with dose-dependent increases in mortality and ESKD risk. Initiating therapy at 7–8 mg/dl was associated with lower 22-year cumulative risks of mortality by –0.76%p (95% CI –0.91 to –0.59) and –0.37%p (–0.44 to –0.29), and ESKD by –1.23%p (–1.44 to –0.98) and –0.43%p (–0.55 to –0.31), estimated to correspond to 100–300 fewer deaths or kidney failure events per 10 000 patients.

**Conclusions:**

Timely initiation of ULT, particularly before serum urate exceeds 9 mg/dl, is associated with improved long-term renal and survival outcomes in CKD. These findings, which may remain undetected in shorter randomized trials, underscore the utility of the g-formula in observational studies as a valuable complement to evidence from randomized trials.

## Graphical Abstract

**Graphical Abstract:**
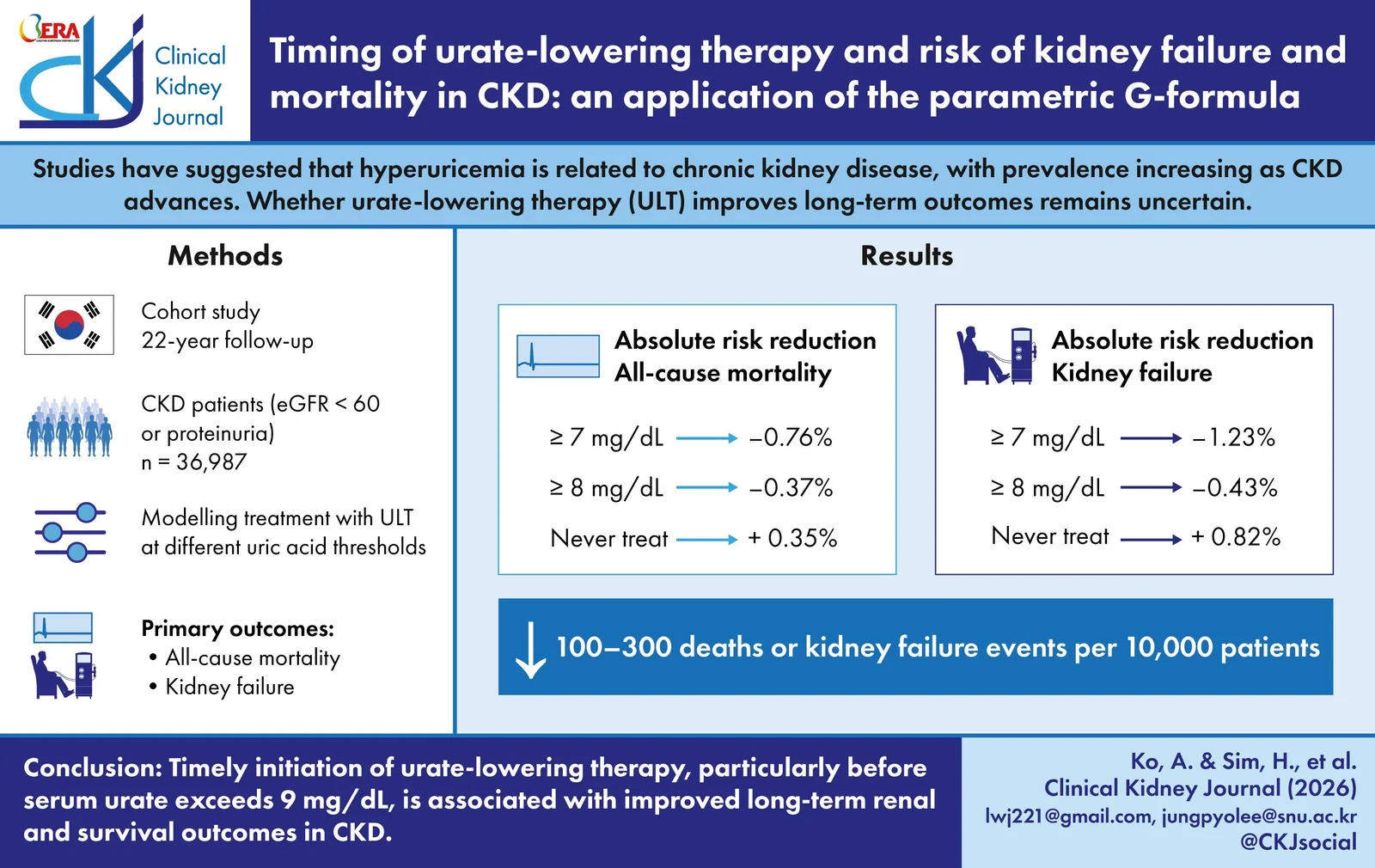

KEY LEARNING POINTS **What was known:**

- Hyperuricemia is common in chronic kidney disease (CKD) and linked to kidney function decline, but whether lowering serum urate improves long-term outcomes remains uncertain.
- Randomized trials of urate-lowering therapy (ULT) have shown neutral results, limited by short follow-up and specific study populations.
- Clinical guidelines differ widely on when to start ULT, leaving treatment decisions largely to physician discretion.

**This study adds:**

- Using two large CKD cohorts and the parametric g-formula, we simulated uniform treatment thresholds while accounting for time-varying confounding.
- Earlier ULT at 7–8 mg/dl was associated with lower long-term risks of kidney failure and mortality compared with later or no treatment.
- Inverse associations were consistent across cohorts and most evident in patients with reduced eGFR, thereby supporting proactive, risk-based initiation strategies.

**This study adds:**

- These findings suggest that timely ULT may be associated with reduced progression to kidney failure and mortality in CKD.
- Results help bridge the gap between short-term randomized trials and real-world clinical practice.
- Incorporating serum urate thresholds and patient risk profiles could guide individualized treatment and clinical decision-making.

## INTRODUCTION

Numerous studies have established that hyperuricemia is closely related to chronic kidney disease (CKD), with its prevalence increasing as CKD advances, regardless of associated symptoms of gout. [1–4] Additionally, hyperuricemia acts as an independent predictor of the onset and progression of CKD, as well as other metabolic diseases. [5–8, 9] Given its socioeconomic burden, addressing modifiable risk factors is crucial to slowing CKD progression. [10, 11]

In this context, the impact of lowering serum uric acid (sUA) concentrations on the prognosis of CKD patients with hyperuricemia has been the subject of research for decades. However, the effects of hyperuricemia treatment on CKD outcomes, especially for those who are asymptomatic, remain unclear. Recent meta-analyses have shown that treating asymptomatic hyperuricemia in CKD patients may slow CKD progression, [12, 13] while others have yielded inconclusive results, [14, 15] and a few have found no benefits. [16] Moreover, certain studies have identified CKD-related benefits only associated with the use of certain urate-lowering agents (ULAs), such as febuxostat. [17, 18] However, current guidelines differ substantially in their recommendations [19–21], leaving uncertainty in daily practice where treatment decisions often rely on physician discretion rather than standardized evidence.

Recently, Johnson *et al*. [22] reported that inconsistent results across studies may reflect differences in study populations and clinical settings, emphasizing the need to identify patient groups that could truly benefit from urate-lowering therapy (ULT). This question motivated recent large-scale randomized controlled trials (RCTs), such as the PERL (Prevention of Early Renal Loss in type 1 Diabetes) and the CKD-FIX (Controlled Trial of Slowing of Kidney Disease Progression from the Inhibition of Xanthine Oxidase) trials, designed to test the efficacy of ULAs in CKD. However, these landmark trials yielded negative results, leaving unresolved questions regarding the optimal target populations and agents. [23, 24] Moreover, given the heterogeneity of CKD patients and the challenges of maintaining long-term follow-up in clinical trials, observational analyses using long-term real-world data remain indispensable complements to RCTs.

To provide additional insights into these unresolved issues, we applied the parametric g-formula, first described by Robins. [25] This method estimates risks under explicitly defined hypothetical interventions while appropriately adjusting for time-varying confounders that are themselves affected by prior treatment. By applying this causal inference framework, we aimed to mitigate potential biases inherent in observational data and approximate the counterfactual data-generating process. [26–28] In this study, we estimated long-term outcomes under different treatment strategies for CKD patients with hyperuricemia, using the g-formula to explore the impact of each strategy.

## MATERIALS AND METHODS

### Study participants and data sources

This study utilized data from two independent cohorts of patients with CKD in South Korea. The main cohort consisted of patients followed at the nephrology clinic of a tertiary medical center (Seoul National University Hospital), while the replication cohort was constructed using the data from a university-affiliated hospital (Seoul National University Boramae Medical Center) to confirm the robustness of our fundings.

From 1 January 2001 to 31 December 2018, a total of 39 938 and 11 440 patients were enrolled in the main and replication cohorts, respectively, and we applied identical eligibility criteria. As in our previous studies [29, 30], we included adults patients with CKD, defined as kidney damage or an eGFR <60 ml/min/1.73 m^2^ for 3 months or more, in accordance with the Kidney Disease: Improving Global Outcomes guidelines [19]. Kidney damage was ascertained by the presence of albuminuria, defined as an albumin-to-creatinine ratio >30 mg/g in two or more spot urine specimens at enrollment. We excluded patients diagnosed with end-stage kidney disease (ESKD) at enrollment, those who experienced an outcome within 3 months of enrollment, and those with ULA prescriptions 30 days before or 90 days after enrollment to exclude prior use (Supplemental Fig. S1).

This study was approved by the institutional review board of Seoul National University Hospital (no. J-2408-007-1557) and Seoul National University Hospital Boramae Medical Center (no. 30-2024-43), and all investigations were conducted in compliance with the Declaration of Helsinki guidelines.

### Data collection and definitions of covariates

Patient information, including demographics, laboratory data, diagnostic history according to the International Classification of Disease 10th Revision (ICD-10) codes, nursing records, and drug prescription history, was collected from electronic medical records (EMRs) in a deidentified manner. The estimated glomerular filtration rate (eGFR) was calculated from Chronic Kidney Disease Epidemiology Collaboration (CKD-EPI) equation using serum creatinine (sCr) concentrations. [31] Comorbidities were defined using ≥2 ICD-10 codes before enrollment (Supplemental Table S1).

### Determination of ULA prescription

In this study, only allopurinol, febuxostat, and benzbromarone were used as ULAs. We defined “ULA prescription status” as “prescribed” if any ULA was taken for longer than 180 cumulative days. The prescribed dosages of each ULA were not considered.

### Outcomes

The primary outcome of the study was defined as all-cause mortality, with mortality data gathered from EMRs and the National Statistical Office of South Korea. The secondary outcome was defined as progression to ESKD, identified when a patient required dialysis (either hemodialysis or peritoneal dialysis) for 3 months or longer, or underwent kidney transplantation. Data on ESKD was sourced from the EMRs and Korean Society of Nephrology Database. The follow-up period was defined as the interval from the first sCr measurement until the occurrence of an interested outcome or the censoring date (31 December 2022, for the main cohort; 31 March 2023, for the replication cohort), whichever came first.

### Statistical methods

We used a target trial framework to conceptualize an ideal study (Supplemental Table S2) [32]. We applied the parametric g-formula, which involved two primary steps. First, using the cohort data from the entire follow-up period, we developed regression models for the time-varying covariates. Second, we simulated time-varying covariates and outcomes under hypothetical interventions based on the estimates from the first step. [27, 28, 33–35]

**Figure 1:**
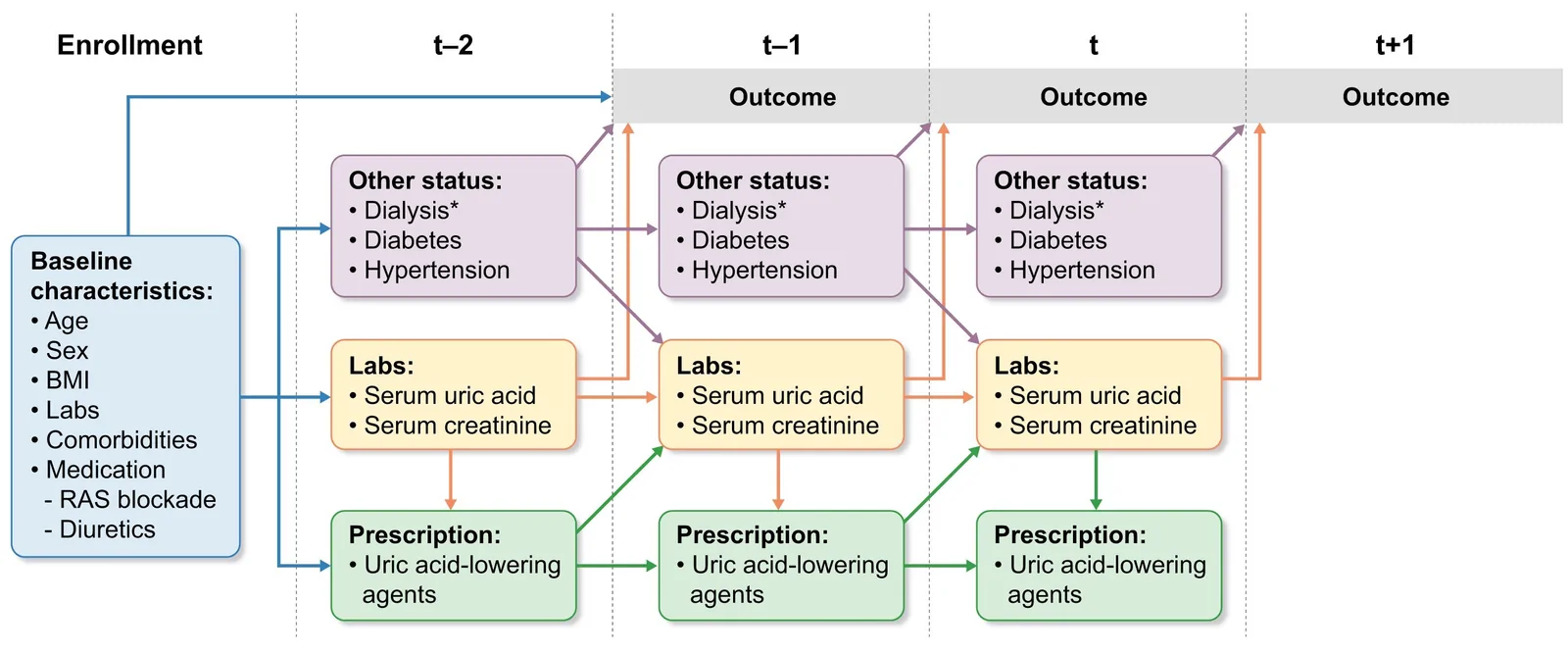
Illustration of dynamics among time-varying covariates in the process of treating hyperuricemia. This figure illustrates the complex dynamics among time-varying covariates in the decision-making process of treating hyperuricemia. When a patient visits the clinic, we mainly check and decide whether to prescribe a drug or not. This decision of prescription is affected by the sCr and sUA concentrations at that time (*t* − 1) and impacts the labs at the next time point (*t)*. In this way, these time-varying confounders affect both outcome and exposure, changing over time. Abbreviations: sCr, serum creatinine; sUA, serum uric acid. *Dialysis status was included as a time-varying covariate only if the outcome is death.

As illustrated in Fig. 1, we modeled the complex dynamics among covariates in the process of hyperuricemia treatment [34]. We created regression models for time-varying confounders (i.e. sUA and sCr concentrations), as well as ULA prescription status and outcomes of interest. Analyses adjusted for time-varying (diabetes, hypertension, and dialysis status) and baseline variables (demographics, labs, and comorbidities). Among comorbidities, only hypertension and diabetes were treated as time-varying covariates, owing to their high prevalence in CKD and established independent impact on progression. Other comorbidities were treated as baseline covariates to reduce model complexity and avoid overfitting. Patients without lab measurements for time-varying covariates (sCr, sUA) over a 24-month period were censored to maintain data integrity. To handle missing values of each covariate, we employed the last observation carried forward method.

Follow-up was restructured into 6-month intervals. At each interval, treatment assignment was modeled conditional on covariates estimated at the immediately preceding time point. We examined six different hypothetical scenarios for managing hyperuricemia. The first scenario reflected observed practice (“real-world practice”), which served as the reference group. [36] We then simulated four threshold-based strategies where ULA therapy was initiated only when sUA concentrations reached 7, 8, 9, or 10 mg/dl, with no treatment provided below these thresholds. The final scenario involved never treating hyperuricemia regardless of sUA concentrations. We simulated the clinical course forward in time by iteratively generating covariates and applying the assigned intervention strategies at each subsequent interval throughout the follow-up period. This process allowed us to estimate the predicted probabilities of outcomes under each strategy. The real-world practice scenario was simulated to emulate the observed data patterns as closely as possible, which was referred to as the “natural course” in the g-formula literature. [28, 35, 36] We calculated the standardized cumulative risk for outcomes using Monte Carlo simulations. [33] The 95% confidence intervals (CIs) were estimated using 1000 bootstrap replicates. To assess the temporal emergence of treatment effects, we calculated cumulative risks at truncated horizons of 5, 10, and 15 years in addition to the full 22-year follow-up.

Effect modification was primarily explored based on eGFR (<60 ml/min/1.73 m² or ≥60 ml/min/1.73 m²), as well as age (<30 years or ≥30 years) and body mass index (BMI) (<25 kg/m², ≥25 kg/m²). For comparative purposes, we conducted traditional time-varying Cox regression analyses. The time-varying Cox models provided supplementary results of the treatment effect, excluding the evolving association with other time-varying confounders. A two-tailed *P* value of 0.05 was considered to indicate statistical significance. All analyses were conducted using R (version 4.3.2, R Core Team 2021, Archive Network: https://cran.r-project.org) [37].

## RESULTS

### Baseline characteristics

The baseline characteristics of the study participants of the main cohort are presented in Table 1. Among a total of 27 260 CKD patients in the main cohort, 5361 used any type of ULA at least once. The mean age of the patients was 51.1 ± 15.9 years, with 49.3% being male. Additionally, 2953 (10.9%) patients had an eGFR ∼45 ml/min/1.73 m^2^ at enrollment. In ULA ever-users, the median UA concentration at the time of ULA initiation was 9.0 mg/dl (1st quantile: 8.0 mg/dl, 3rd quantile: 10.2 mg/dl), and the median duration from enrollment to ULA initiation was 5 years (1st quantile: 2 years, 3rd quantile: 10 years). Compared to nonusers, ULA ever-users had a higher proportion of males and comorbidities such as diabetes mellitus and hypertension. The median follow-up duration was 10.6 years for ESKD and 14.9 years for mortality, and during this period, 3290 patients developed ESKD, and 5560 patients died.

**Table 1:** Baseline characteristics of the cohort: comparison of ULA ever-users and non-users.

	Ever-user(*n* = 5361)	Non-user(*n* = 21 899)	*P*-value	Missing proportion (%)
Age, year	52.43 ± 15.88	50.78 ± 15.86	<0.001	0
Sex, male, No. (%)	3752 (69.99)	9342 (42.66)	<0.001	0
BMI, kg/m^2^, No. (%)			<0.001	31
18.5−	210 (3.92)	605 (2.76)		–
18.5–23	1377 (25.69)	4974 (22.71)		–
23–25	1153 (21.51)	3372 (15.40)		–
25–30	1701 (31.73)	4404 (20.11)		–
30–35	237 (4.42)	594 (2.71)		–
35+	54 (1.01)	122 (0.56)		–
**Comorbidity**				
Diabetic Mellitus, No. (%)	2067 (38.56)	5554 (25.36)	<0.001	0
Hypertension, No. (%)	3760 (70.14)	9271 (42.34)	<0.001	0
Dyslipidemia, No. (%)	1603 (29.90)	5439 (24.84)	<0.001	0
Myocardial infarction, No. (%)	46 (0.86)	88 (0.40)	<0.001	0
Chronic heart failure, No. (%)	49 (0.91)	95 (0.43)	<0.001	0
Peripheral vascular disease, No. (%)	57 (1.06)	122 (0.56)	<0.001	0
Cerebral vascular disease, No. (%)	217 (4.05)	584 (2.67)	<0.001	0
Liver disease, No. (%)	300 (5.6)	1004 (4.58)	0.002	0
Cancer, No. (%)	444 (8.28)	1537 (7.02)	0.002	0
**Laboratory findings**				
White blood cell, ×10^3^/μl	6.92 ± 2.66	6.47 ± 2.38	<0.001	17
Hemoglobin, mg/dl	13.25 ± 2.28	13.33 ± 1.93	0.01	13
C-reactive protein, mg/dl	0.93 ± 3.21	0.74 ± 3.00	0.01	64
Albumin, g/dl	3.98 ± 0.56	4.13 ± 0.51	<0.001	6
Glucose, mg/dl	120.17 ± 58.22	110.22 ± 45.88	<0.001	6
Total bilirubin, mg/dl	0.84 ± 1.25	0.82 ± 0.72	0.09	6
Total cholesterol, mg/dl	194.6 ± 56.15	194.03 ± 51.82	0.49	6
Protein, mg/dl	7.14 ± 0.79	7.24 ± 0.69	<0.001	6
Creatinine, mg/dl	1.5 ± 0.92	1.12 ± 0.84	<0.001	0
eGFR, ml/min/1.73m^2^			<0.001	0
Stage				
1–2	2615 (48.78)	17 252 (78.78)		–
3a	1257 (23.45)	2463 (11.25)		–
3b	867 (16.17)	1130 (5.16)		–
4	495 (9.23)	732 (3.34)		–
5	127 (2.37)	322 (1.47)		–
Uric acid, mg/dl	7.58 ± 2.20	5.58 ± 1.81	<0.001	0

ULA ever-user refers to individuals who have used any type of ULA at least once during the follow-up period. Values for continuous variables given as mean ± standard deviation. Abbreviations: ULA, uric acid-lowering agent; BMI, body mass index; eGFR, estimated glomerular filtration rate; and ESKD, end-stage kidney disease.

**Table 2:** Simulated risk estimates for all-cause mortality and ESKD under hypothetical intervention by the g-formula.

		Risk (%)		Risk ratio (95% CI)	Risk difference (95% CI)
Outcome	Intervention	Observed	by the G-formula		*(% points)*
Primary outcome: **All-cause mortality**	Real-world practice *(no intervention)*	25.6	23.6	Reference	Reference
	Never-treating regardless of sUA	–	23.9	1.015 (1.009, 1.020)	0.35 (0.21, 0.47)
	Treating if sUA ≥10 mg/dl; otherwise, no treatment	–	23.7	1.007 (1.002, 1.011)	0.15 (0.05, 0.26)
	Treating if sUA ≥9 mg/dl; otherwise, no treatment	–	23.5	0.998 (0.994, 1.001)	−0.05 (−0.13, 0.02)
	Treating if sUA ≥8 mg/dl; otherwise, no treatment	–	23.2	0.984 (0.981, 0.988)	−0.37 (−0.44, −0.29)
	Treating if sUA ≥7 mg/dl; otherwise, no treatment	–	22.8	0.968 (0.962, 0.975)	−0.76 (−0.91, −0.59)
Secondary outcome: **ESKD**	Real-world practice *(no intervention)*	20.5	19.6	Reference	Reference
	Never-treating regardless of sUA	–	20.5	1.042 (1.023, 1.056)	0.82 (0.45, 1.10)
	Treating if sUA ≥10 mg/dl; otherwise, no treatment	–	20.2	1.027 (1.013, 1.039)	0.53 (0.24, 0.76)
	Treating if sUA ≥9 mg/dl; otherwise, no treatment	–	19.8	1.009 (0.998, 1.018)	0.17 (−0.04, 0.36)
	Treating if sUA ≥8 mg/dl; otherwise, no treatment	–	19.2	0.978 (0.972, 0.985)	−0.43 (−0.55, −0.31)
	Treating if sUA ≥7 mg/dl; otherwise, no treatment	–	18.4	0.937 (0.927, 0.949)	−1.23 (−1.44, −0.98)

Real-world practice represented “no intervention”. The risk estimates by the g-formula under the real-world practice scenario closely matched the observed risks from the original data for both outcomes, indicating the model’s validity. Risk ratios are presented to three decimal places and risk differences to two decimal places to ensure clarity regarding statistical significance. Abbreviations: ESKD, end-stage kidney disease; sUA, serum uric acid.

### Parametric g-formula results of the main cohort

Table 2 summarizes the risk estimates under the hypothetical treatment scenarios. Model-simulated risks closely matched observed data, demonstrating good calibration (Supplemental Figs S2a–b). [38] The simulated 22-year cumulative risks of all-cause mortality and ESKD by the g-formula were 23.6% and 19.6%, respectively. The never-treating scenario was associated with greater risks of all-cause mortality and ESKD compared to both real-world practice and treatment strategies based on sUA concentration thresholds. Additionally, initiating treatment at lower sUA thresholds was associated with progressively lower risks of both outcomes.

**Figure 2:**
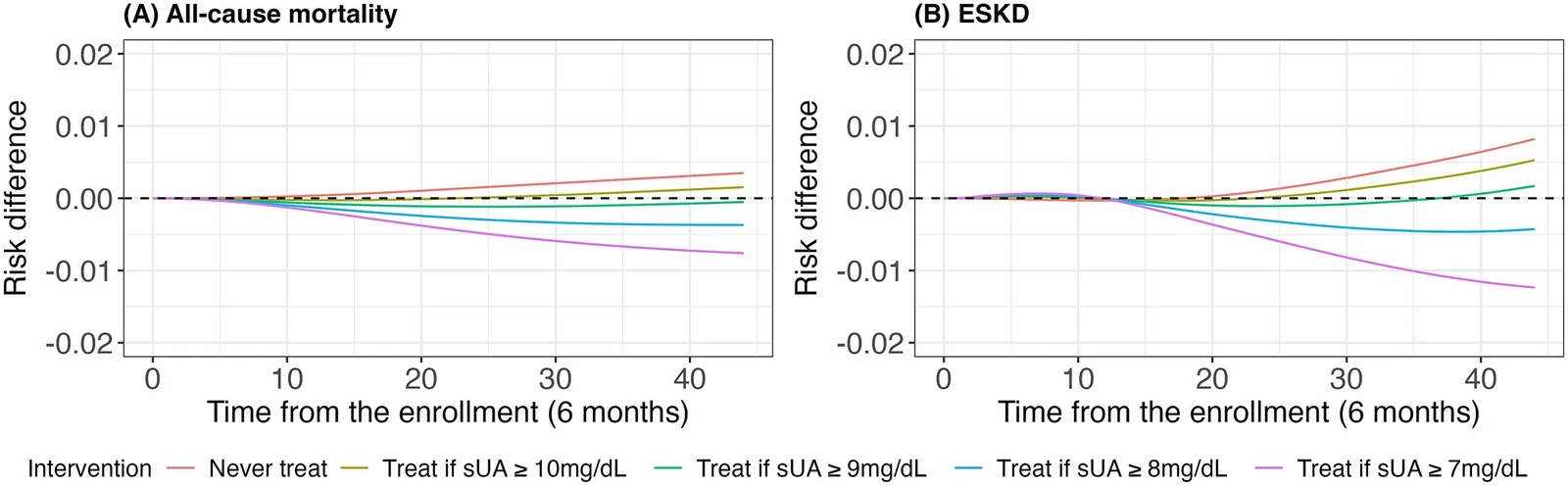
The cumulative risk differences of outcomes compared to the real-world practice scenario by the g-formula. Over time, risk differences between intervention strategies became more pronounced, with the risk difference for treatment initiation at 7 and 8 mg/dl consistently remaining negative throughout the entire follow-up period, indicating a sustained long-term benefit relative to current clinical practice. However, for interventions initiated at higher sUA thresholds, the risk difference became positive, highlighting the risks of delayed treatment. The *x*-axis represents follow-up in 6-month intervals (e.g. 10 = 5 years, 20 = 10 years). Abbreviation: ESKD, end-stage kidney disease.

Compared to the real-world practice, delaying treatment or omitting it entirely was associated with an increasing risk for both outcomes over time (Fig. 2). When evaluated using the 22-year cumulative risk, the RD for all-cause mortality when treatment was initiated at an sUA concentration of ≥10 mg/dl was 0.15%p (95% CI: 0.05–0.26) and in the never-treating scenario was 0.35%p (95% CI: 0.21–1.47). A significant risk reduction for mortality risk was observed at 8 and 7 mg/dl, estimated to correspond to ∼101 and 207 fewer deaths in our cohort of 27 260 CKD patients [RD −0.37%p (95% CI: −0.44 to −0.29) and RD −0.76%p (95% CI: −0.91 to −0.59), respectively]. A reduction in risk was also noted at 9 mg/dl; however, this association did not reach statistical significance.

Significantly lower risks were also observed when treatment was initiated at sUA concentrations of 8 and 7 mg/dl [RD −0.43%p (95% CI: −0.55 to −0.31) and RD −1.23%p (95% CI: −1.44 to −0.98), respectively], estimated to correspond to ∼117 and 335 fewer cases of ESKD in our cohort. In contrast, delaying treatment until an sUA concentration exceeded 9 mg/dl, or never treating, was associated with greater ESKD risk: 9 mg/dl [RD 0.17%p (95% CI: −0.04−0.36)], 10 mg/dl [RD 0.53%p (95% CI: 0.24−0.76)], and the never-treating scenario [RD 0.82%p (95% CI: 0.45–1.10)]. A similar trend was observed in RR estimates across both outcomes.

Considering the median follow-up durations of each outcome, we estimated cumulative risks at 5, 10, and 15 years. ESKD risk differences were nonsignificant within 5 years, consistent with prior RCTs. However, with longer follow-up horizons, the pattern became more pronounced: higher sUA thresholds were associated with increasing risk, while lower thresholds showed lower risks for ESKD. For mortality, a similar trend was observed, with initiating treatment at 7 or 8 mg/dl consistently associated with reduced risks across all truncation points, whereas further delays or omission of treatment were associated with either a very small difference or an increase in risk (Fig. 2 and Supplemental Table S3).

### Subgroup analyses

**Figure 3:**
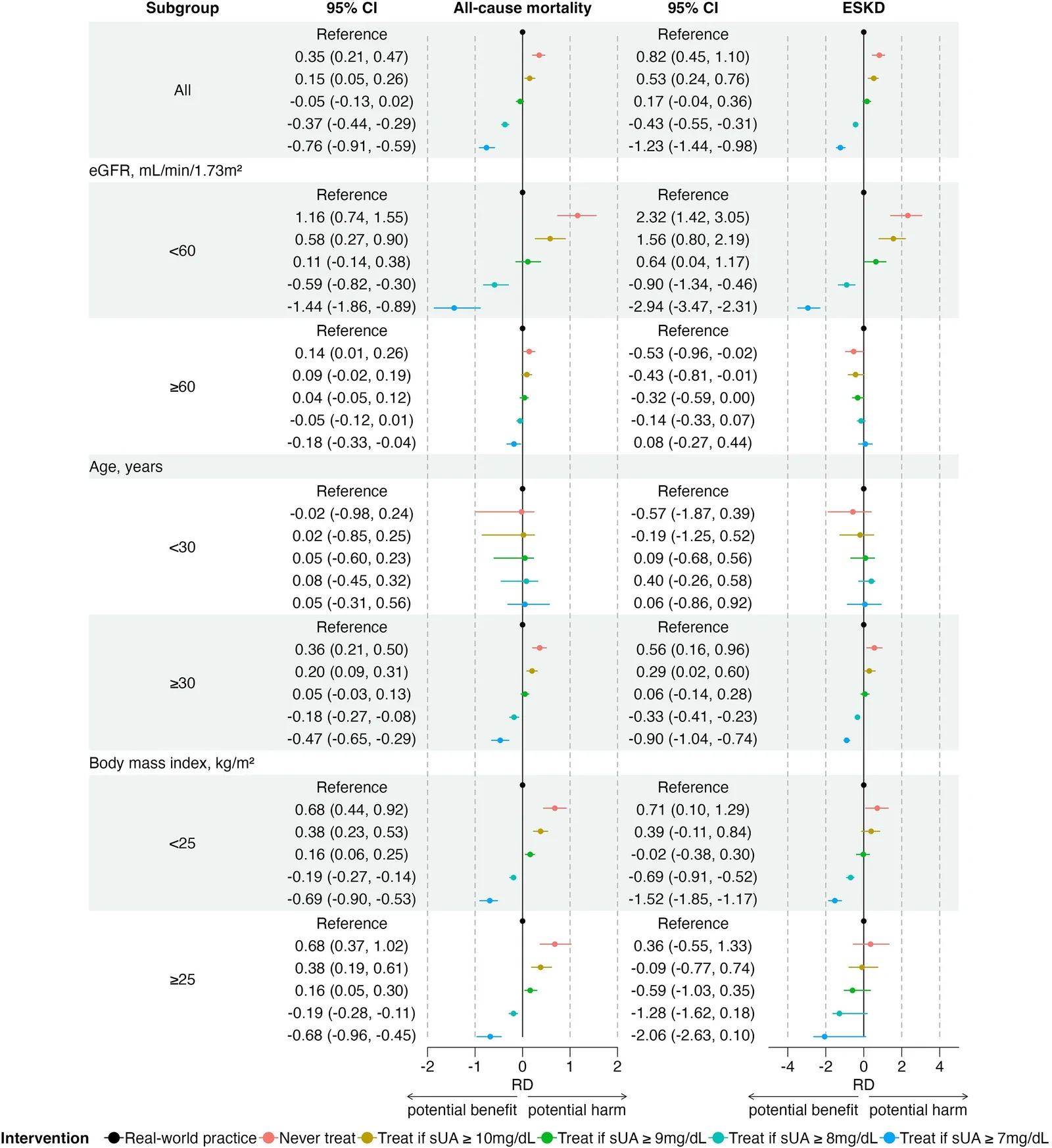
Forest plot of risk differences for outcomes in the entire group and subgroups: comparison of the impact of initiating hyperuricemia treatment when sUA concentration reached specific thresholds vs. the real-world practice. In subgroup analysis, the real-world practice scenario served as the reference, reflecting current clinical practices where treatment is initiated based on physician’s decision. Abbreviations: sUA, serum uric acid; ESKD, end-stage kidney disease; and eGFR, estimated glomerular filtration rate.

Subgroup analyses stratified by eGFR, age, and BMI (Supplemental Tables S6–S7) showed broadly consistent trends. Patients with eGFR <60 ml/min/1.73 m², age ≥30 years, or BMI <25 kg/m² had higher absolute risks for both outcomes. Across nearly all subgroups except those aged <30 years, delaying or omitting treatment was linked to increased mortality and ESKD risks, whereas initiating therapy at 7–8 mg/dl showed the most favorable outcomes. The magnitude of this estimated reduction was most pronounced in lower eGFR and lower BMI groups, corresponding to several hundred fewer deaths or ESKD events in our cohort (Fig. 3 and Supplemental Tables S5–S6).

### Replication cohort analysis using the g-formula

A total of 9727 patients were included in the final analysis among the replication cohort (Supplemental Fig. S1B). Similar to the main cohort, the g-formula model demonstrated good calibration in the replication cohort, as the simulated risks under the natural course closely tracked the observed risks (Supplemental Fig. S2c–d). The g-formula produced similar cumulative risk patterns, with higher risks when treatment was deferred or omitted and consistent inverse associations at 7–8 mg/dl (Supplemental Fig. S3). Subgroup analyses confirmed results largely concordant with the main cohort, reinforcing robustness across settings (data not shown).

## DISCUSSION

By modeling different initiation thresholds for ULT, we examined how treatment timing may influence CKD outcomes using the parametric g-formula. Our findings indicated that initiating hyperuricemia treatment at sUA concentrations of 7 or 8 mg/dl was associated with lower risks of both mortality and ESKD, whereas delaying treatment to higher thresholds was linked to less favorable outcomes. These findings suggest that optimizing the timing of intervention based on sUA concentrations could help inform clinical decision-making regarding initiation timing rather than relying on heterogeneous practices.

A key rationale for employing the g-formula in this study was to evaluate multiple long-term treatment strategies, a task that is practically challenging in RCTs due to the logistical difficulties of enrolling and retaining vulnerable subjects over decades. Unlike traditional time-varying Cox models, which may yield inconsistent results due to residual confounding from the feedback loop between treatments and clinical status (Supplemental Table S8), the g-formula explicitly models these dynamics, reflecting real-world decision-making. [27, 39] Through this robust modeling approach, our results suggest that treating hyperuricemia—whether guided by clinician’s decision (i.e. the real-world practice) or uniform criteria—may be preferable to not treating at all. Although this differs from guidelines recommending treatment only for symptomatic cases [19, 21], our results highlight the potential value of considering broader therapeutic approaches while acknowledging the need for careful patient selection. Notably, our study provided consistent findings regarding all-cause mortality, with risks increasing when treatment was postponed until sUA exceeded higher thresholds or when omitted entirely.

Two landmark RCTs (PERL and CKD-FIX) tested allopurinol in CKD but found no survival or renal benefits despite sustained sUA reduction. [23, 24] Similarly, our 5–10 years estimates showed little evidence of benefit, but protective associations became apparent with extended follow-up beyond 10 years, suggesting that the potential benefits of ULT may require prolonged exposure or broader CKD representation. This divergence may reflect differences in baseline renal function and inclusion criteria, as most RCT participants had advanced or diabetic CKD [40], as well as the use of a single agent and high dropout rates due to safety concerns, which may have led to an underestimation of efficacy. [22] Together, these observations illustrate how the g-formula can complement trial evidence by providing insights into long-term and population-wide associations not easily assessed in short-term RCTs.

Similarly, the FEATHER trial and several smaller studies reported overall neutral results but observed benefit in selected subgroups, particularly those with preserved renal function and minimal proteinuria. [41–43] In contrast, our subgroup analyses indicated that patients with an eGFR <60 ml/min/1.73 m² or age ≥30 year showed consistent associations with lower risk when ULT was initiated at lower sUA thresholds, particularly below 8–9 mg/dl. In contrast, those with preserved kidney function or younger age showed minimal risk differences across thresholds. These findings suggest that treatment decisions might benefit from being stratified by baseline renal risk rather than applying uniform thresholds, and that unnecessary intervention in very low-risk patients should be approached with caution.

Experimental studies have demonstrated that hyperuricemia can trigger inflammation and endothelial dysfunction, promoting renal fibrosis and a self-perpetuating cycle in CKD. Initial increases in sUA can elevate blood pressure, while chronic conditions, such as reduced nephron numbers, play a role in maintaining high blood pressure and its sequelae. [22, 44–46] Therefore, early identification of the suitable timing and patient groups for hyperuricemia treatment is crucial, especially given that the sUA concentrations linked to increased risk of total and cardiovascular mortality—4.7 and 5.6 mg/dl, respectively [47]—suggesting that the thresholds for adverse outcomes in CKD may be considerably lower than the current clinical definitions of hyperuricemia.

Like other causal inference methods, the g-formula relies on assumptions of no unmeasured confounding, accurate measurement, and correct model specification. It is sensitive to violations, as errors can propagate through the analysis. [28, 34] Specifically, unmeasured factors such as medication adherence or detailed lifestyle habits (e.g. diet) could not be fully captured in this EMR-based dataset. Although residual bias cannot be excluded, our estimates under the real-world practice scenario closely predicted observed values, supporting model calibration. [38] We adjusted for potential confounders and performed sensitivity analyses, but did not compare the effectiveness of individual ULAs or other urate-lowering drugs. [40]

In summary, our findings suggest that timely initiation of ULT—particularly before sUA exceeds 9 mg/dl—is associated with improved renal and survival outcomes in CKD. By leveraging long-term follow-up and simulating uniform treatment strategies, our study revealed potential long-term benefits that may remain undetected in shorter trials, particularly among patients with eGFR <60 ml/min/1.73 m² or aged ≥30 years. Moreover, this study underscores the value of the g-formula as a tool for pharmacoepidemiologic research using observational data, enabling robust estimation of treatment strategies while serving as a complement to evidence from randomized trials.

## Supplementary Material

sfag103_Supplemental_File

## Data Availability

Data will be made available on reasonable request.
