# Correction to: Timing of urate-lowering therapy and risk of kidney failure and mortality in CKD: an application of the parametric G-formula

**DOI:** 10.1093/ckj/sfag308

**Published:** 2026-09-21

This is a correction to: Ara Ko, Hyunman Sim, Soie Kwon, Seung Hyun Han, Ali Abu-Alfa, Dong Ki Kim, Chun Soo Lim, Jung Pyo Lee, Woojoo Lee, Timing of urate-lowering therapy and risk of kidney failure and mortality in CKD: an application of the parametric G-formula, *Clinical Kidney Journal*, Volume 19, Issue 5, May 2026, sfag103, https://doi.org/10.1093/ckj/sfag103.

In the originally published version of this manuscript, an affiliation was omitted for co-author Hyunman Sim.

The following affiliation has been added: “Department of Statistics, Jeonbuk National University, Jeonju, Republic of Korea”.

All subsequent affiliations have been re-numbered accordingly.

