# Supplementary material for: Timing of urate-lowering therapy and risk of kidney failure and mortality in CKD: an application of the parametric G-formula
Source: Clin Kidney J. 2026 Mar 23;19(5):sfag103. doi: 10.1093/ckj/sfag103 (PMC13133626; doi:10.1093/ckj/sfag103)
Supplement: sfag103_Supplemental_File [file sfag103_supplemental_file.docx]

**Supplementary appendix**

**Table of Contents**

**Figure S1.** Cohort derivation flow chart (A) of the main cohort and (B) of the replication cohort.

**Figure S2.** Model calibration: Comparison of observed and simulated cumulative risks under the real-world practice scenario.

**Figure S3.** Forest plot of risk differences for outcomes in the replication cohort.

**Table S1.** ICD-10 codes definitions of adjusted comorbidities in this study.

**Table S2.** Defining the hypothetical target trial for hyperuricemia treatment in CKD patients and its simulation using the g-formula.

**Table S3.** Cumulative risks truncated at 5, 10, and 15 years.

**Table S4.** Baseline characteristics of the validation cohort.

**Table S5.** Simulated risk estimates for outcomes in the replication cohort under hypothetical intervention by the g-formula.

**Table S6.** Subgroup analysis results in the main cohort for all-cause mortality, stratified by (A) eGFR, (B) age, and (C) BMI

**Table S7.** Subgroup analysis results in the main cohort for ESKD, stratified by (A) eGFR, (B) age, and (C) BMI

**Table S8.** Results of time-varying Cox regression analysis for comparative purposes.

**Figure S1A. Cohort derivation flow chart**


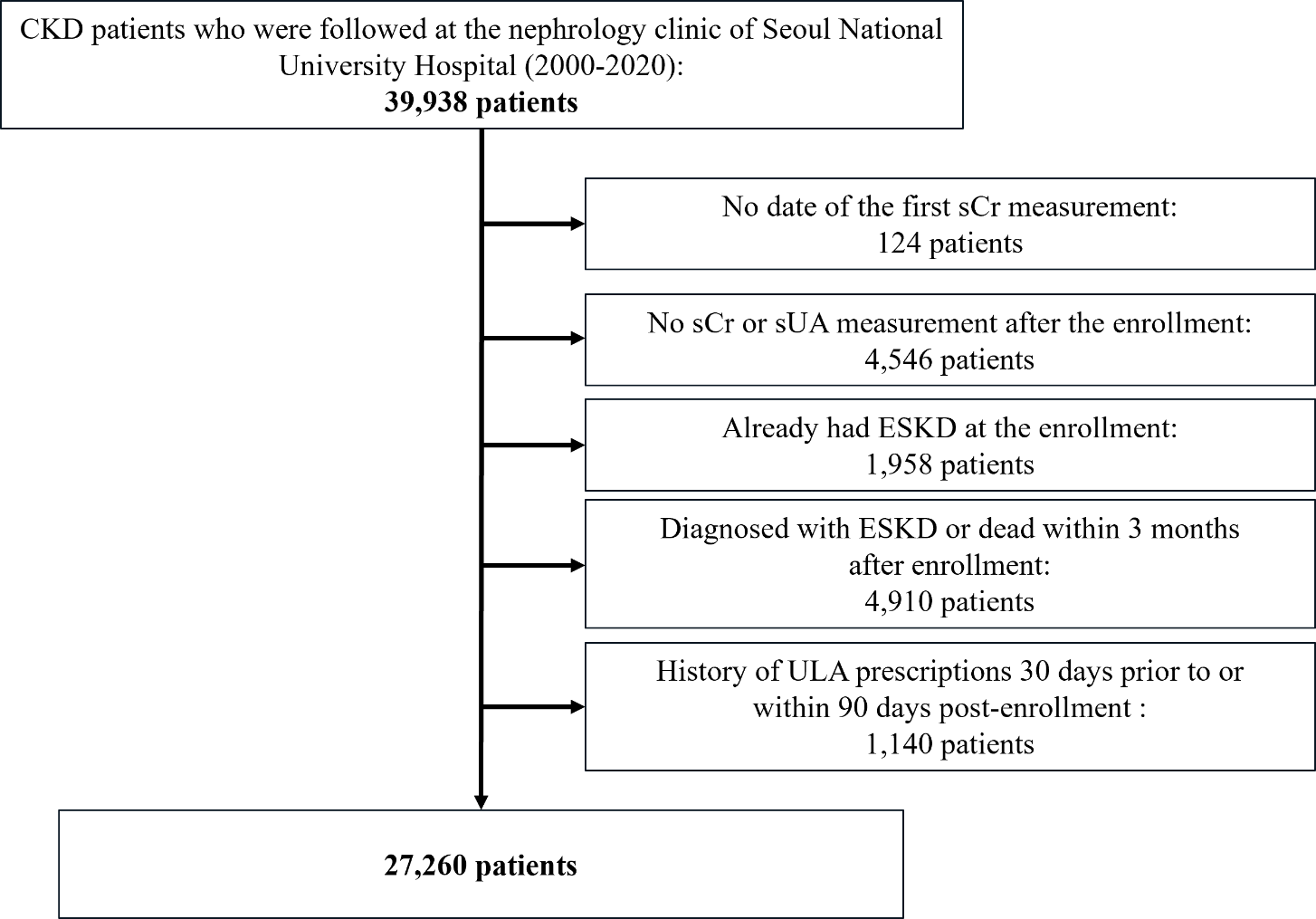


Abbreviations: sCr, serum creatinine; sUA, serum uric acid; ESKD, end-stage kidney disease; ULA, uric-acid lowering agent.

**Figure S1B. Cohort derivation flow chart of the replication cohort**


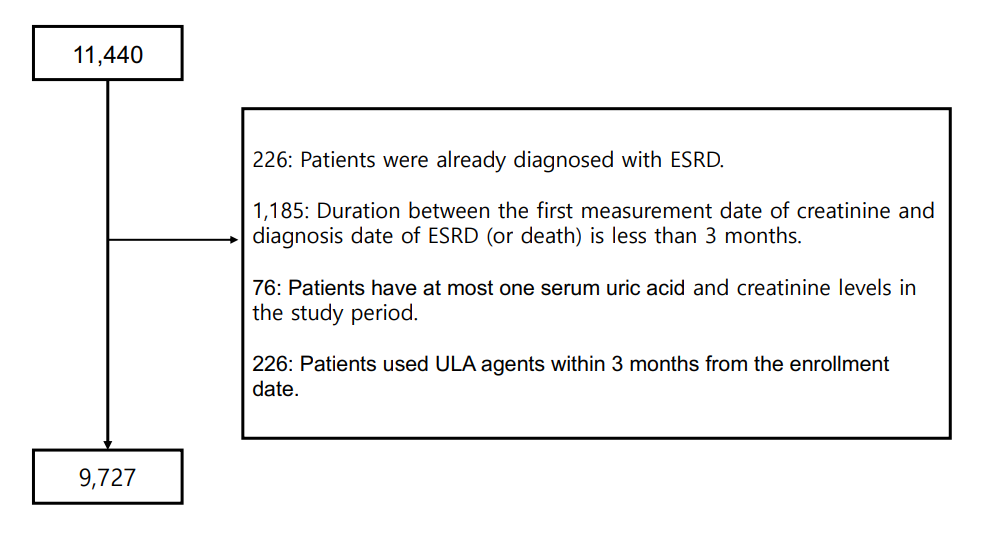


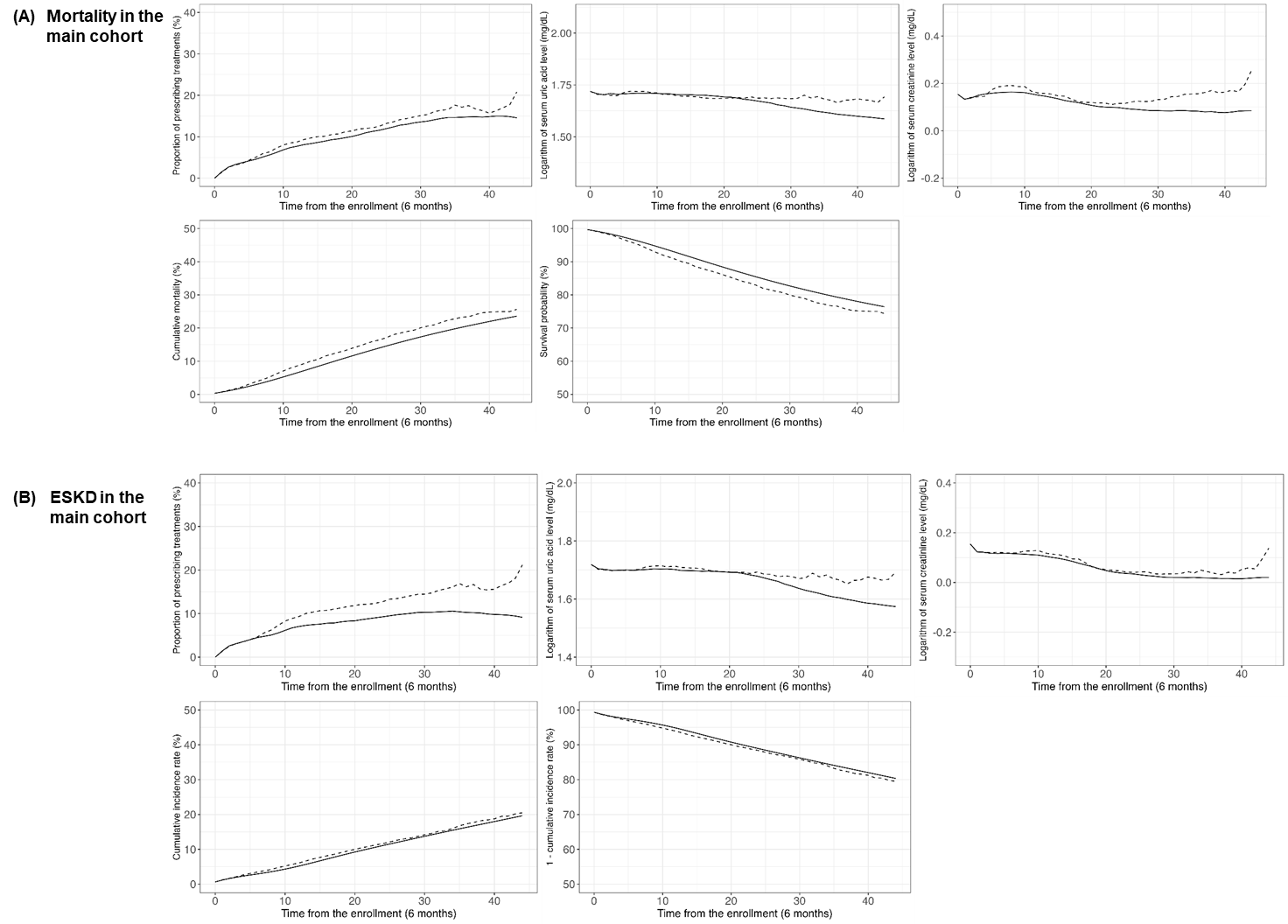
**Figure S2. Model calibration: Comparison of observed and simulated cumulative risks under the real-world practice scenario.**


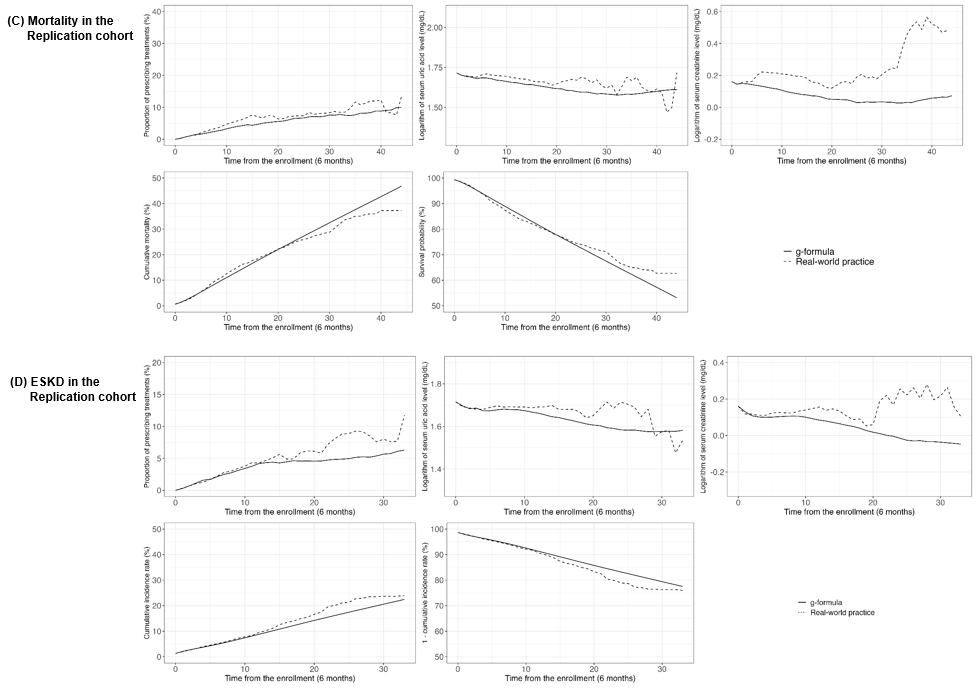


(A) All-cause mortality and (B) ESKD in the Main cohort; (C) All-cause mortality and (D) ESKD in the Replication cohort. In each panel, the top three figures compare the observed and simulated estimates for the time-varying covariates: proportion of ULA prescription, sUA level, and sCr level. The bottom two figures compare the observed and simulated cumulative incidence (or mortality) and survival probability (or 1 - cumulative incidence). Solid lines represent the predicted values estimated using the g-formula, while dashed lines indicate the observed values from real-world data. **Abbreviations:** ULA, urate-lowering agent; sUA, serum uric acid; sCr, serum creatinine; ESKD, end-stage kidney disease.


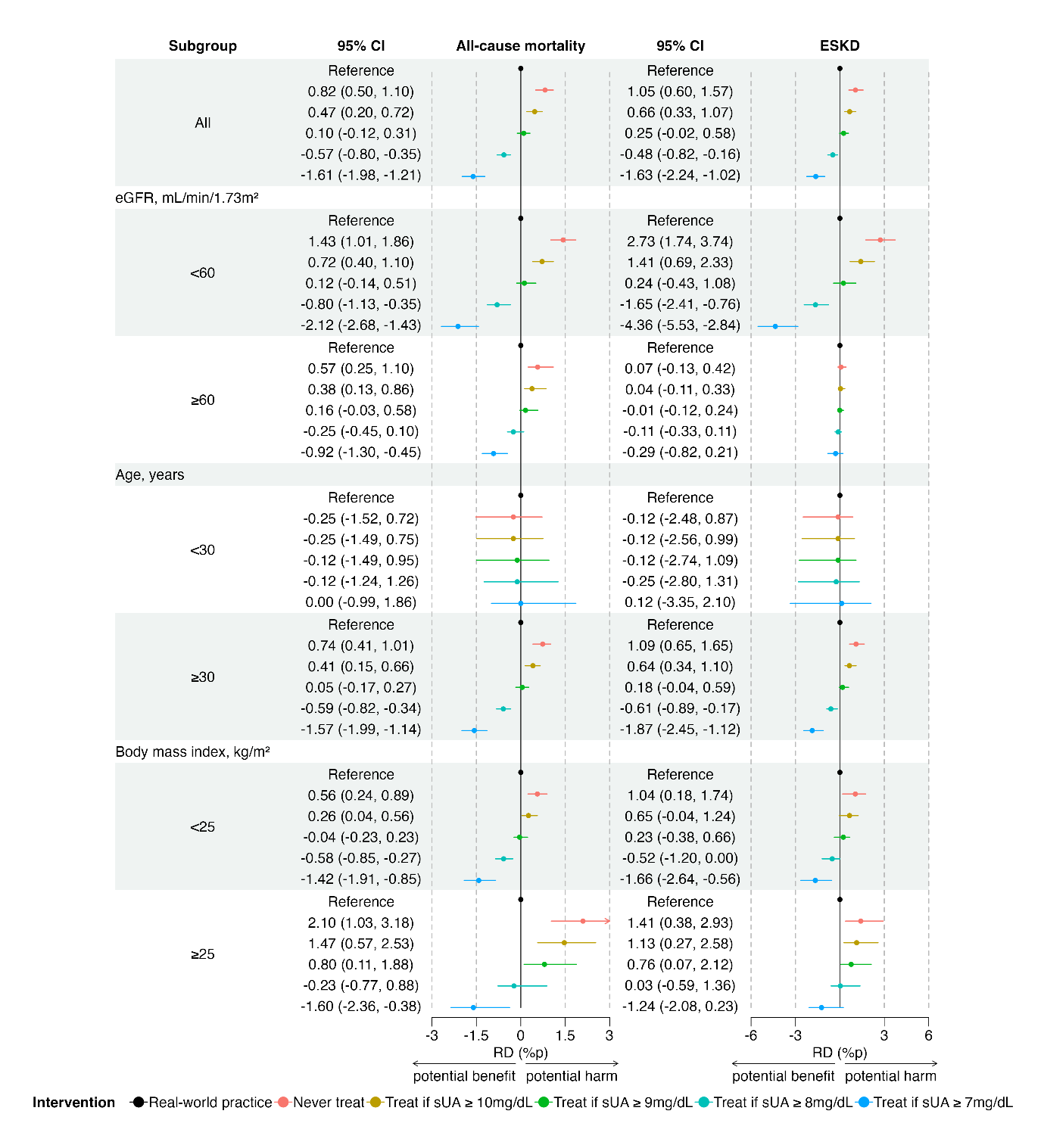
**Figure S3.** **Forest plot of risk differences for outcomes in the replication cohort.**

As in the main cohort, the real-world practice scenario served as the reference, reflecting current clinical practices where treatment is initiated based on physician’s decision. Abbreviations: sUA, serum uric acid; ESKD, end-stage kidney disease; eGFR, estimated glomerular filtration rate.

**Table S1. ICD-10 codes definitions of adjusted comorbidities in this study.**

| **Comorbidity** | **ICD-10 code definitions** |
| --- | --- |
| Diabetes | E10.x-E14.x |
| Hypertension | I10.x-I13.x, I15.x |
| Dyslipidemia | E78.x |
| Chronic heart failure | I11.0, I13.0, I13.2, I27.x, I28.0, I42.x, I23.x, I50.x, I51.5, I51.7, I52.8, Z94.1, Z94.3 |
| Cerebrovascular disease | I60.x-I69.x |
| Peripheral vascular disease | I73.x, I79.2, I79.8, I70.2, I73.1, I73.8 |
| Liver disease | K70.x-K77.x |
| Malignancy | C code |

Abbreviations: ICD-10, International Classification of Disease 10^th^ Revision.

**Table S2.** **Defining the hypothetical target trial for hyperuricemia treatment in CKD patients and its simulation using the g-formula.**

| **Element** | **Description** | **Hypothetical target trial** | **Simulation using observational data with the g-formula** |
| --- | --- | --- | --- |
| Eligibility  criteria | Who will be included in this study? | Individuals ≥18 years with CKD (i.e., kidney damage or an eGFR <60 mL/min per 1.73 m^2^ for 3 months or more), baseline data on sUA and sCr concentrations, followed for at least 3 months at nephrology clinics, and no use of any ULA in the previous 30 days between January 2001 and December 2018. Excludes patients with prior ESKD or outcomes within the first 3 months to ensure stable follow-up. | Same as target trial, excluding patients with ULA prescriptions 30 days prior to or within 90 days post-enrollment to ensure washout. |
| Treatment  strategies | Which precise treatment strategies or interventions will eligible individuals receive? | 1. Initiate ULA treatment only when sUA concentrations reach or exceed specified thresholds (≥7 mg/dL, ≥8 mg/dL, ≥9 mg/dL, or ≥10 mg/dL), and withhold treatment when sUA concentrations are below these thresholds. 2. Never treat regardless of sUA concentrations. | 1. Initiate ULA treatment uniformly at specified sUA thresholds (≥7 mg/dL, ≥8 mg/dL, ≥9 mg/dL, or ≥10 mg/dL), and withhold treatment when sUA concentrations fall below these thresholds. 2. Never treat regardless of sUA concentrations 3. Follow real-world practice as closely emulated using g-formula-derived estimates. |
| Treatment  assignment | How will eligible individuals be assigned to the treatment strategies? | Random allocation to treatment strategies based on sUA thresholds, no blinding | ULA prescription status is deterministically assigned based on dynamically simulated sUA concentrations (*i.e.*, if the sUA concentration exceeds the predefined threshold, the status is set to ‘prescribed’; otherwise, it is set to ‘not prescribed’), strictly adhering to treatment decisions modeled on predefined sUA thresholds throughout the follow-up period. |
| Follow-up  period | When does follow-up start and when does it end? | Starts at randomization and ends at occurrence of end point, or end of study (December 31, 2022). | Same as target trial, but starting at the first sCr measurement instead. For data completeness, patients with no measurements for time-varying covariates (sCr, sUA) over a 24-month period were censored in the modeling process. |
| Outcomes | What outcomes will be measured during follow-up? | 1. All-cause mortality 2. Progression to ESKD (dialysis for 3 months or more, or kidney transplantation) | Same as target trial; outcomes captured through EMRs and national databases. |
| Causal estimand | Which causal estimand will be estimated with the observational data? | Intention-to-treat effect (effect of treatment assignment by sUA thresholds, regardless of adherence)  Per protocol effect (effect among individuals fully adhering to treatment assignment) | Average treatment effect estimated by simulating predicted outcomes for all eligible patients under each intervention strategy, with adjustment for time-varying confounders to improve causal inference accuracy. |
| Statistical  analysis | Which statistical analyses will be used to estimate the causal estimand? | Intention-to-treat analysis and per protocol analysis | Predict time-varying covariates and outcomes for each intervention by modeling throughout the follow-up, ensuring confounding is controlled dynamically. |

This table outlines the parallels and distinctions between the idealized target trial and our g-formula-based simulation. This approach enabled us to analyze the effects of various hypothetical treatment strategies for hyperuricemia in situations where conducting RCTs may not be feasible. Abbreviations:

**Table S3. Cumulative risks truncated at 5, 10, and 15 years.**

| **Outcome** | **Intervention** | **Risk** | **Risk ratio (95% CI)** | **Risk difference (95% CI)** |
| --- | --- | --- | --- | --- |
|  |  | *(%)* |  | *(% points)* |
| **All-cause mortality** | **5-year mark** | | | |
|  | Real-world practice | 5.2 | **1** | **0** |
|  | Never-treating regardless of sUA | 5.3 | 1.00 (1.00, 1.01) | 0.03 (0.02, 0.03) |
|  | Treating if sUA ≥10 mg/dL;  otherwise, no treatment | 5.2 | 1.00 (0.99, 1.00) | -0.02 (-0.03, -0.02) |
|  | Treating if sUA ≥9 mg/dL;  otherwise, no treatment | 5.2 | 0.99 (0.99, 0.99) | -0.06 (-0.07, -0.04) |
|  | Treating if sUA ≥8 mg/dL;  otherwise, no treatment | 5.1 | 0.98 (0.98, 0.99) | -0.10 (-0.13, -0.07) |
|  | Treating if sUA ≥7 mg/dL;  otherwise, no treatment | 5.1 | 0.98 (0.97, 0.98) | -0.13 (-0.17, -0.08) |
|  | **10-year mark** | | | |
|  | Real-world practice | 11.6 | 1 | 0 |
|  | Never-treating regardless of sUA | 11.7 | 1.01 (1.01, 1.01) | 0.10 (0.07, 0.14) |
|  | Treating if sUA ≥10 mg/dL;  otherwise, no treatment | 11.6 | 1.00 (1.00, 1.00) | -0.01 (-0.03, 0.01) |
|  | Treating if sUA ≥9 mg/dL;  otherwise, no treatment | 11.5 | 0.99 (0.99, 0.99) | -0.11 (-0.13, -0.08) |
|  | Treating if sUA ≥8 mg/dL;  otherwise, no treatment | 11.3 | 0.98 (0.97, 0.98) | -0.24 (-0.29, -0.18) |
|  | Treating if sUA ≥7 mg/dL;  otherwise, no treatment | 11.2 | 0.97 (0.96, 0.98) | -0.38 (-0.47, -0.26) |
|  | **15-year mark** | | | |
|  | Real-world practice | 17.3 | 1 | 0 |
|  | Never-treating regardless of sUA | 17.5 | 1.01 (1.01, 1.02) | 0.21 (0.13, 0.28) |
|  | Treating if sUA ≥10 mg/dL;  otherwise, no treatment | 17.4 | 1.00 (1.00, 1.01) | 0.04 (-0.01, 0.10) |
|  | Treating if sUA ≥9 mg/dL;  otherwise, no treatment | 17.2 | 0.99 (0.99, 1.00) | -0.11 (-0.14, -0.07) |
|  | Treating if sUA ≥8 mg/dL;  otherwise, no treatment | 17.0 | 0.98 (0.98, 0.98) | -0.34 (-0.39, -0.26) |
|  | Treating if sUA ≥7 mg/dL;  otherwise, no treatment | 16.7 | 0.97 (0.96, 0.97) | -0.59 (-0.71, -0.43) |
| **ESKD** | **5-year mark** | | | |
|  | Real-world practice | 4.3 | 1 | 0 |
|  | Never-treating regardless of sUA | 4.3 | 0.99 (0.99, 1.00) | -0.03 (-0.05, -0.01) |
|  | Treating if sUA ≥10 mg/dL;  otherwise, no treatment | 4.3 | 1.00 (1.00, 1.00) | 0.00 (-0.01, 0.01) |
|  | Treating if sUA ≥9 mg/dL;  otherwise, no treatment | 4.3 | 1.00 (1.00, 1.01) | 0.02 (-0.01, 0.04) |
|  | Treating if sUA ≥8 mg/dL;  otherwise, no treatment | 4.4 | 1.01 (1.00, 1.02) | 0.03 (-0.01, 0.06) |
|  | Treating if sUA ≥7 mg/dL;  otherwise, no treatment | 4.4 | 1.01 (1.00, 1.02) | 0.04 (-0.02, 0.10) |
|  | **10-year mark** | | | |
|  | Real-world practice | 9.2 | 1 | 0 |
|  | Never-treating regardless of sUA | 9.3 | 1.00 (0.99, 1.01) | 0.03 (-0.06, 0.12) |
|  | Treating if sUA ≥10 mg/dL;  otherwise, no treatment | 9.2 | 1.00 (0.99, 1.00) | -0.03 (-0.08, 0.03) |
|  | Treating if sUA ≥9 mg/dL;  otherwise, no treatment | 9.1 | 0.99 (0.99, 0.99) | -0.10 (-0.14, -0.06) |
|  | Treating if sUA ≥8 mg/dL;  otherwise, no treatment | 9.0 | 0.98 (0.97, 0.99) | -0.22 (-0.29, -0.13) |
|  | Treating if sUA ≥7 mg/dL;  otherwise, no treatment | 8.9 | 0.96 (0.95, 0.98) | -0.36 (-0.50, -0.20) |
|  | **15-year mark** | | | |
|  | Real-world practice | 13.8 | 1 | 0 |
|  | Never-treating regardless of sUA | 14.0 | 1.02 (1.01, 1.03) | 0.28 (0.09, 0.44) |
|  | Treating if sUA ≥10 mg/dL;  otherwise, no treatment | 13.9 | 1.01 (1.00, 1.02) | 0.11 (-0.01, 0.23) |
|  | Treating if sUA ≥9 mg/dL;  otherwise, no treatment | 13.7 | 0.99 (0.99, 1.00) | -0.08 (-0.17, 0.00) |
|  | Treating if sUA ≥8 mg/dL;  otherwise, no treatment | 13.3 | 0.97 (0.96, 0.98) | -0.41 (-0.49, -0.31) |
|  | Treating if sUA ≥7 mg/dL;  otherwise, no treatment | 12.9 | 0.94 (0.93, 0.96) | -0.82 (-0.99, -0.59) |

Real-world practice represented ‘no intervention’. Results are reported to four decimal places to ensure precision and avoid including 1 in RR or 0 in RD confidence intervals. Abbreviations: ESKD, end-stage kidney disease; sUA, serum uric acid.

**Table S4.** **Baseline characteristics of the replication cohort**

|  | **Ever-user**  **(*n* = 486)** | **Non-user**  **(*n* = 9,241)** | **P-value** | **Missing proportion (%)** |
| --- | --- | --- | --- | --- |
| Age, year | 61.12 ± 14.59 | 57.17 ± 16.99 | < 0.001 | 0 |
| Sex, male, No. (%) | 103 (21.19) | 5297 (57.32) | < 0.001 | 0 |
| BMI, kg/m^2^, No. (%) |  |  | < 0.001 | 35 |
| 18.5- | 36 (7.41) | 484 (5.24) |  | - |
| 18.5-23 | 126 (25.93) | 2102 (22.75) |  | - |
| 23-25 | 84 (17.28) | 1245 (13.47) |  | - |
| 25-30 | 129 (26.54) | 1685 (18.23) |  | - |
| 30-35 | 20 (4.12) | 311 (3.37) |  | - |
| 35+ | 5 (1.03) | 52 (0.56) |  | - |
| **Comorbidity** | | | | |
| Diabetic Mellitus, No. (%) | 212 (44.17) | 3063 (33.3) | < 0.001 | 1 |
| Hypertension, No. (%) | 340 (71.28) | 4511 (49.11) | < 0.001 | 1 |
| Dyslipidemia, No. (%) | 167 (34.36) | 2793 (30.22) | 0.06 | 0 |
| Myocardial infarction, No. (%) | 23 (4.73) | 207 (2.24) | 0.001 | 0 |
| Chronic heart failure, No. (%) | 80 (16.46) | 701 (7.59) | < 0.001 | 0 |
| Peripheral vascular disease, No. (%) | 35 (7.2) | 376 (4.07) | 0.001 | 0 |
| Cerebral vascular disease, No. (%) | 147 (30.25) | 1855 (20.07) | < 0.001 | 0 |
| Liver disease, No. (%) | 78 (16.05) | 1047 (11.33) | 0.002 | 0 |
| Cancer, No. (%) | 125 (25.72) | 1445 (15.64) | < 0.001 | 0 |
| **Laboratory findings** | | | | |
| White blood cell, x10^3^/μL | 7.9 ± 2.67 | 7.69 ± 3.41 | 0.19 | 9 |
| Hemoglobin, mg/dL | 13.27 ± 2.22 | 12.91 ± 2.09 | < 0.001 | 8 |
| C-reactive protein, mg/dL | 1.82 ± 3.38 | 3.29 ± 6.93 | 0.03 | 73 |
| Albumin, g/dL | 3.92 ± 0.47 | 3.97 ± 0.47 | 0.07 | 10 |
| Glucose, mg/dL | 140.21 ± 95.84 | 131.83 ± 72.46 | 0.02 | 5 |
| Total cholesterol, mg/dL | 187.63 ± 53.59 | 187.01 ± 50.49 | 0.81 | 8 |
| Protein, mg/dL | 7.01 ± 0.75 | 6.98 ± 0.72 | 0.51 | 10 |
| Creatinine, mg/dL | 1.96 ± 1.82 | 1.49 ± 1.6 | < 0.001 | 0 |
| eGFR, mL/min/1.73m^2^ |  |  | < 0.001 | 0 |
| Stage |  |  |  |  |
| 1-2 | 128 (26.34) | 5682 (61.49) |  | - |
| 3a | 126 (25.93) | 1200 (12.99) |  | - |
| 3b | 127 (26.13) | 1070 (11.58) |  | - |
| 4 | 87 (17.9) | 815 (8.82) |  | - |
| 5 | 18 (3.7) | 474 (5.13) |  | - |
| Uric acid, mg/dL | 7.7 ± 2.03 | 5.42 ± 1.92 | < 0.001 | 5 |

ULA ever-user refers to individuals who have used any type of ULA at least once during the follow-up period. Values for continuous variables given as mean ± standard deviation. Abbreviations: ULA, uric acid-lowering agent; BMI, body mass index; eGFR, estimated glomerular filtration rate; ESKD, end-stage kidney disease.

**Table S5.** **Simulated risk estimates for outcomes in the replication cohort under hypothetical intervention by the g-formula**

| **Outcome** | **Intervention** | **Risk** *(%)* | | **Risk ratio (95% CI)** | **Risk difference (95% CI)** |
| --- | --- | --- | --- | --- | --- |
|  |  | **Observed** | **by the G-formula** |  | *(% points)* |
| Primary outcome: **All-cause mortality** | Real-world practice | 37.3 | 47.9 | Reference | Reference |
|  | Never-treating regardless of sUA | - | 48.7 | 1.017 (1.011, 1.023) | 0.82 (0.50, 1.10) |
|  | Treating if sUA ≥10 mg/dL;  otherwise, no treatment | - | 48.4 | 1.010 (1.004, 1.015) | 0.47 (0.20, 0.72) |
|  | Treating if sUA ≥9 mg/dL;  otherwise, no treatment | - | 48.0 | 1.002 (0.997, 1.006) | 0.10 (-0.12, 0.31) |
|  | Treating if sUA ≥8 mg/dL;  otherwise, no treatment | - | 47.3 | 0.988 (0.983, 0.993) | -0.57 (-0.80, -0.35) |
|  | Treating if sUA ≥7 mg/dL;  otherwise, no treatment | - | 46.3 | 0.966 (0.958, 0.975) | -1.61 (-1.98, -1.21) |
| Secondary outcome: **ESKD** | Real-world practice | 24.0 | 23.1 | Reference | Reference |
|  | Never-treating regardless of sUA | - | 24.1 | 1.046 (1.028, 1.061) | 1.05 (0.60, 1.57) |
|  | Treating if sUA ≥10 mg/dL;  otherwise, no treatment | - | 23.7 | 1.029 (1.016, 1.041) | 0.66 (0.33, 1.07) |
|  | Treating if sUA ≥9 mg/dL;  otherwise, no treatment | - | 23.3 | 1.011 (0.999, 1.023) | 0.25 (-0.02, 0.58) |
|  | Treating if sUA ≥8 mg/dL;  otherwise, no treatment | - | 22.6 | 0.979 (0.963, 0.994) | -0.48 (-0.82, -0.16) |
|  | Treating if sUA ≥7 mg/dL;  otherwise, no treatment | - | 21.4 | 0.929 (0.903, 0.957) | -1.63 (-2.24, -1.02) |

The risk estimates by the g-formula under the real-world practice scenario closely matched the observed risks from the original data for both outcomes, indicating the model's good calibration. Risk ratios are presented to three decimal places and risk differences to two decimal places to ensure clarity regarding statistical significance. Abbreviations: ESKD, end-stage kidney disease; sUA, serum uric acid.

**Table S6. Subgroup analysis results in the main cohort for all-cause mortality, stratified by (A) eGFR, (B) age, and (C) BMI**

| **Intervention** | **Estimated risk  by the g-formula** | | **Risk ratio (95% CI)** | | **Risk difference (95% CI)** | |
| --- | --- | --- | --- | --- | --- | --- |
|  | *(%)* | |  | | *(% points)* | |
| **(A)** | **eGFR (mL/min/1.73m²) at baseline** | | | | | |
|  | **< 60** | **≥ 60** | **< 60** | **≥ 60** | **< 60** | **≥ 60** |
| Real-world practice | 52.9 | 16.7 | Reference | Reference | Reference | Reference |
| Never-treating regardless of sUA | 54.1 | 16.8 | 1.022 (1.013, 1.030) | 1.008 (1.000, 1.016) | 1.16 (0.74, 1.55) | 0.14 (0.01, 0.26) |
| Treating if sUA ≥10 mg/dL;  otherwise, no treatment | 53.5 | 16.7 | 1.011 (1.005, 1.017) | 1.006 (0.999, 1.012) | 0.58 (0.27, 0.90) | 0.09 (-0.02, 0.19) |
| Treating if sUA ≥9 mg/dL;  otherwise, no treatment | 53.0 | 16.7 | 1.002 (0.997, 1.007) | 1.003 (0.997, 1.007) | 0.11 (-0.14, 0.38) | 0.04 (-0.05, 0.12) |
| Treating if sUA ≥8 mg/dL;  otherwise, no treatment | 52.3 | 16.6 | 0.989 (0.984, 0.995) | 0.997 (0.993, 1.001) | -0.59 (-0.82, -0.30) | -0.05 (-0.12, 0.01) |
| Treating if sUA ≥7 mg/dL;  otherwise, no treatment | 51.5 | 16.5 | 0.973 (0.963, 0.984) | 0.989 (0.980, 0.998) | -1.44 (-1.86, -0.89) | -0.18 (-0.33, -0.04) |
| **(B)** | **Age (years) at baseline** | | | | | |
|  | **< 30** | **≥ 30** | **< 30** | **≥ 30** | **< 30** | **≥ 30** |
| Real-world practice | 6.6 | 23.8 | Reference | Reference | Reference | Reference |
| Never-treating regardless of sUA | 6.5 | 24.1 | 0.996 (0.953, 1.046) | 1.015 (1.009, 1.021) | -0.02 (-0.98, 0.24) | 0.36 (0.21, 0.50) |
| Treating if sUA ≥10 mg/dL;  otherwise, no treatment | 6.6 | 24.0 | 1.003 (0.958, 1.039) | 1.009 (1.004, 1.013) | 0.02 (-0.85, 0.25) | 0.20 (0.09, 0.31) |
| Treating if sUA ≥9 mg/dL;  otherwise, no treatment | 6.6 | 23.8 | 1.008 (0.955, 1.029) | 1.002 (0.999, 1.006) | 0.05 (-0.60, 0.23) | 0.05 (-0.03, 0.13) |
| Treating if sUA ≥8 mg/dL;  otherwise, no treatment | 6.7 | 23.6 | 1.012 (0.929, 1.044) | 0.993 (0.989, 0.996) | 0.08 (-0.45, 0.32) | -0.18 (-0.27, -0.08) |
| Treating if sUA ≥7 mg/dL;  otherwise, no treatment | 6.6 | 23.3 | 1.007 (0.919, 1.080) | 0.980 (0.972, 0.988) | 0.05 (-0.31, 0.56) | -0.47 (-0.65, -0.29) |
| **(C)** | **BMI (kg/m^2^) at baseline** | | | | | |
|  | **< 25** | **≥ 25** | **< 25** | **≥ 25** | **< 25** | **≥ 25** |
| Real-world practice | 24.1 | 19.7 | Reference | Reference | Reference | Reference |
| Never-treating regardless of sUA | 24.7 | 20.4 | 1.028 (1.019, 1.038) | 1.034 (1.019, 1.049) | 0.68 (0.44, 0.92) | 0.68 (0.37, 1.02) |
| Treating if sUA ≥10 mg/dL;  otherwise, no treatment | 24.4 | 20.1 | 1.016 (1.010, 1.021) | 1.020 (1.010, 1.029) | 0.38 (0.23, 0.53) | 0.38 (0.19, 0.61) |
| Treating if sUA ≥9 mg/dL;  otherwise, no treatment | 24.2 | 19.9 | 1.007 (1.003, 1.010) | 1.008 (1.003, 1.014) | 0.16 (0.06, 0.25) | 0.16 (0.05, 0.30) |
| Treating if sUA ≥8 mg/dL;  otherwise, no treatment | 23.9 | 19.5 | 0.992 (0.989, 0.994) | 0.991 (0.985, 0.995) | -0.19 (-0.27, -0.14) | -0.19 (-0.28, -0.11) |
| Treating if sUA ≥7 mg/dL;  otherwise, no treatment | 23.4 | 19.0 | 0.971 (0.963, 0.978) | 0.965 (0.952, 0.977) | -0.69 (-0.90, -0.53) | -0.68 (-0.96, -0.45) |

As in the main result, real-world practice represented ‘no intervention’. Risk ratios are presented to three decimal places and risk differences to two decimal places to ensure clarity regarding statistical significance. Abbreviations: sUA, serum uric acid; BMI, body mass index; eGFR, estimated glomerular filtration rate.

**Table S7.** **Subgroup analysis results in the main cohort for ESKD, stratified by (A) eGFR, (B) age, and (C) BMI**

| **Intervention** | **Estimated risk  by the g-formula** | | **Risk ratio (95% CI)** | | **Risk difference (95% CI)** | |
| --- | --- | --- | --- | --- | --- | --- |
|  | *(%)* | |  | | *(% points)* | |
| **(A)** | **eGFR (mL/min/1.73m²) at baseline** | | | | | |
|  | **< 60** | **≥ 60** | **< 60** | **≥ 60** | **< 60** | **≥ 60** |
| Real-world practice | 64.9 | 15.4 | Reference | Reference | Reference | Reference |
| Never-treating regardless of sUA | 67.2 | 14.8 | 1.036 (1.022, 1.047) | 0.966 (0.932, 0.998) | 2.32 (1.42, 3.05) | -0.53 (-0.96, -0.02) |
| Treating if sUA ≥10 mg/dL;  otherwise, no treatment | 66.5 | 14.9 | 1.024 (1.012, 1.034) | 0.972 (0.942, 0.999) | 1.56 (0.80, 2.19) | -0.43 (-0.81, -0.01) |
| Treating if sUA ≥9 mg/dL;  otherwise, no treatment | 65.5 | 15.0 | 1.010 (1.001, 1.018) | 0.979 (0.958, 1.000) | 0.64 (0.04, 1.17) | -0.32 (-0.59, 0.00) |
| Treating if sUA ≥8 mg/dL;  otherwise, no treatment | 64.0 | 15.2 | 0.986 (0.979, 0.993) | 0.991 (0.976, 1.004) | -0.90 (-1.34, -0.46) | -0.14 (-0.33, 0.07) |
| Treating if sUA ≥7 mg/dL;  otherwise, no treatment | 62.0 | 15.4 | 0.955 (0.946, 0.965) | 1.005 (0.983, 1.030) | -2.94 (-3.47, -2.31) | 0.08 (-0.27, 0.44) |
| **(B)** | **Age (years) at baseline** | | | | | |
|  | **< 30** | **≥ 30** | **< 30** | **≥ 30** | **< 30** | **≥ 30** |
| Real-world practice | 20.4 | 22.4 | Reference | Reference | Reference | Reference |
| Never-treating regardless of sUA | 19.8 | 23.0 | 0.972 (0.874, 1.022) | 1.025 (1.007, 1.043) | -0.57 (-1.87, 0.39) | 0.56 (0.16, 0.96) |
| Treating if sUA ≥10 mg/dL;  otherwise, no treatment | 20.2 | 22.7 | 0.991 (0.916, 1.029) | 1.013 (1.001, 1.027) | -0.19 (-1.25, 0.52) | 0.29 (0.02, 0.60) |
| Treating if sUA ≥9 mg/dL;  otherwise, no treatment | 20.5 | 22.5 | 1.005 (0.957, 1.032) | 1.003 (0.994, 1.012) | 0.09 (-0.68, 0.56) | 0.06 (-0.14, 0.28) |
| Treating if sUA ≥8 mg/dL;  otherwise, no treatment | 20.8 | 22.1 | 1.019 (0.990, 1.029) | 0.986 (0.982, 0.990) | 0.40 (-0.26, 0.58) | -0.33 (-0.41, -0.23) |
| Treating if sUA ≥7 mg/dL;  otherwise, no treatment | 20.4 | 21.5 | 1.003 (0.954, 1.054) | 0.960 (0.954, 0.966) | 0.06 (-0.86, 0.92) | -0.90 (-1.04, -0.74) |
| **(C)** | **BMI (kg/m^2^) at baseline** | | | | | |
|  | **< 25** | **≥ 25** | **< 25** | **≥ 25** | **< 25** | **≥ 25** |
| Real-world practice | 29.7 | 23.4 | Reference | Reference | Reference | Reference |
| Never-treating regardless of sUA | 30.4 | 23.8 | 1.024 (1.004, 1.043) | 1.016 (0.968, 1.064) | 0.71 (0.10, 1.29) | 0.36 (-0.55, 1.33) |
| Treating if sUA ≥10 mg/dL;  otherwise, no treatment | 30.1 | 23.3 | 1.013 (0.996, 1.028) | 0.996 (0.960, 1.038) | 0.39 (-0.11, 0.84) | -0.09 (-0.77, 0.74) |
| Treating if sUA ≥9 mg/dL;  otherwise, no treatment | 29.7 | 22.8 | 0.999 (0.987, 1.010) | 0.975 (0.953, 1.016) | -0.02 (-0.38, 0.30) | -0.59 (-1.03, 0.35) |
| Treating if sUA ≥8 mg/dL;  otherwise, no treatment | 29.0 | 22.1 | 0.977 (0.969, 0.983) | 0.945 (0.925, 1.017) | -0.69 (-0.91, -0.52) | -1.28 (-1.62, 0.18) |
| Treating if sUA ≥7 mg/dL;  otherwise, no treatment | 28.2 | 21.3 | 0.949 (0.938, 0.959) | 0.912 (0.878, 1.014) | -1.52 (-1.85, -1.17) | -2.06 (-2.63, 0.10) |

As in the main result, real-world practice represented ‘no intervention’. Risk ratios are presented to three decimal places and risk differences to two decimal places to ensure clarity regarding statistical significance. Abbreviations: ESKD, end-stage kidney disease; sUA, serum uric acid; BMI, body mass index; eGFR, estimated glomerular filtration rate.

**Table S8. Results of time-varying Cox regression analysis for comparative purposes**

| **Outcome** | **HR** | **95% CI** | ***p* value** |
| --- | --- | --- | --- |
| **All-cause mortality** | 0.91 | 0.80-1.02 | 0.1 |
| **ESKD** | 2.17 | 1.95-2.41 | <0.0001 |

The time-varying Cox models were used for comparative purposes, incorporating the same covariates as the g-formula, and only applied to the main cohort. Abbreviations: ESKD, end-stage kidney disease.
